# Donor-Related Risk Factors for Graft Decompensation Following Descemet's Stripping Automated Endothelial Keratoplasty

**DOI:** 10.3389/fmed.2022.810536

**Published:** 2022-02-04

**Authors:** Sota Nishisako, Takefumi Yamaguchi, Masatoshi Hirayama, Kazunari Higa, Dai Aoki, Chiaki Sasaki, Hisashi Noma, Jun Shimazaki

**Affiliations:** ^1^Cornea Center and Eye Bank, Tokyo Dental College, Ichikawa General Hospital, Chiba, Japan; ^2^Department of Ophthalmology, Tokyo Dental College, Ichikawa General Hospital, Chiba, Japan; ^3^Department of Data Science, The Institute of Statistical Mathematics, Tokyo, Japan

**Keywords:** donor-related risk factors, donor pseudophakic lens status, preoperative endothelial folds, Descemet's stripping automated endothelial keratoplasty (DSAEK), non-fuchs' endothelial corneal dystrophy patients

## Abstract

**Aims:**

To identify donor-related risk factors associated with graft endothelial failure and postoperative endothelial cell density (ECD) reduction after Descemet's stripping automated endothelial keratoplasty (DSAEK).

**Methods:**

This was a single-center retrospective study conducted from July 2006-December 2016. We included 584 consecutive eyes (482 patients) that underwent DSAEK for the treatment of laser iridotomy-related bullous keratopathy (192 eyes), pseudophakic bullous keratopathy (137 eyes), regraft (96 eyes), Fuchs' endothelial corneal dystrophy (FECD; 59 eyes) and others (100 eyes). Twenty-three donor- and recipient-related risk factors potentially associated with graft failure and ECD reduction were assessed using Cox hazard models and linear mixed effect models.

**Results:**

The median age of the patients was 73.5 years (male; 35.6%). After DSAEK, ECD decreased from 2,674 cells/mm^2^ (95% confidence interval [CI]; 2,646–2,701) to 1,132 (1,076–1,190) at 12 months and 904 (845–963) at 24 months (*P* < 0.001). Fifty-five eyes (9.4%) had graft endothelial failure without rejection. This failure was associated with donor pseudophakic lens status (hazard ratio [HR]; 2.67, CI; 1.50–4.76, *P* = 0.001) and preoperative endothelial folds (HR; 2.82, CI; 1.20–6.62, *P* = 0.02). The incidence of graft endothelial failure in non-FECD patients was significantly higher among those receiving donor grafts with a pseudophakic lens status and preoperative presence of endothelial folds (*P* < 0.001). Postoperative ECD loss was significantly greater in eyes with these risk factors compared to those without (*P* = 0.007).

**Conclusions:**

Pseudophakic status and/or presence of preoperative endothelial folds are the significant donor risk factors for endothelial failure in non-FECD patients.

## Introduction

Corneal endothelial dysfunction is one of the leading causes of blindness among patients with corneal diseases (1). Selective replacement of the damaged corneal endothelium by Descemet's stripping automated endothelial keratoplasty (DSAEK) or Descemet's membrane endothelial keratoplasty (DMEK) allow rapid visual recovery (2, 3), resistance to trauma, and minimum astigmatism in comparison with conventional penetrating keratoplasty (PKP), and were able to improve the prognosis of corneal transplantation for bullous keratopathy (BK) eyes (4–6). However, late corneal endothelial failure owing to chronic loss of corneal endothelial cells remains a clinically relevant issue that should be addressed to improve the long-term prognosis of endothelial keratoplasty (7).

Previous clinical studies have shown various risk factors, including donor and recipient factors, associated with endothelial cell density (ECD) loss and graft failure (8–13). However, these results for donor risk factors after DSAEK were obtained in the United States or Europe, where Fuchs' endothelial corneal dystrophy (FECD) is the most common indication in recipient cohorts. In contrast, the indications for DSAEK in Asian countries are different: 70% of endothelial keratoplasties in these countries were performed for treating pseudophakic bullous keratopathy (PBK) or laser-iridotomy-related bullous keratopathy (LIBK), and the number of cases involving FECD accounted for approximately 10% of all transplants (14–16). Although a thorough assessment of donor-related risk factors to improve graft survival after DSAEK is essential, the data for cases involving non-FECD corneal edema disease in Asian countries have been scarce. Therefore, this study aimed to evaluate donor-related factors that may be associated with corneal endothelial failure and reduction in ECD after DSAEK, especially for recipients with non-FECD.

## Materials and Methods

This study was conducted according to the tenets of the Declaration of Helsinki, and it received approval from the Institutional Ethics Reviewer Board of Tokyo Dental College Ichikawa General Hospital (Acceptance No. I 18-19). Our Institutional Review Board waived the requirement for informed consent for this retrospective study. Patient data were anonymized before access and/or analysis.

### Data Collection and Analysis

From July 2006 through December 2016, 587 eyes of 485 patients who underwent DSAEK at the Tokyo Dental College Ichikawa General Hospital were enrolled. All eligible donor corneas met the medical standards of the Eye Bank Association of America or Cornea Center and Eye Bank (CCEB, Chiba, Japan). All domestic corneas were donated to Japanese eye banks and were transported to Tokyo Dental College Ichikawa General Hospital via CCEB. Imported corneas were prepared at an eye bank in the United States of America (Sight-Life, Seattle, WA) and were shipped internationally by airplane. All donor corneas were preserved in a viewing storage chamber and kept in cold-storage corneal preservation medium (Optisol-GS solution; Bausch and Lomb Surgical, Rochester, NY, USA) at a temperature of 2°-8°C. In the eye bank, the central corneal ECD of all tissues was measured using specular microscopy and slit-lamp microscopy was performed by eye bank technicians or eye doctors to evaluate transplant suitability (i.e., epithelial/stromal/endothelial cells/endothelial folds/cutting issue). ECDs of all the grafts used for DSAEK in this study were more than 2,000 cells/mm^2^ preoperatively. The DSAEK procedure was performed using the double-glide technique in a standardized manner (17, 18). Briefly, Descemet stripping was performed using a reverse-bent Sinsky hook (ASICO, Westmont, IL), and the recipient's endothelium and Descemet's membrane were carefully removed with forceps. A precut donor tissue was trephinated, and was gently inserted into the anterior chamber using the Busin glide spatula (ASICO). Air was carefully injected into the anterior chamber to unfold the graft. Ten minutes after the air injection, half of the air was replaced with a balanced salt solution (Alcon, Fort Worth, TX). Postoperatively, topical 0.1% betamethasone (Sanbetazon; Santen, Osaka, Japan) qid was prescribed for 6 months. Six months after DSAEK, 0.1% fluorometholone (Flumetholone 0.1; Santen) was prescribed three times a day for up to 12 months after surgery. ECD was measured by blinded orthoptists at 1, 3, 6, 12, and 24 months after DSAEK using a specular microscopy system (EM-4000; TOMEY, Nagoya, Japan). Approximately 50 cells were analyzed for mean ECD (17). In some patients, direct ECD measurement was difficult due to corneal edema or interface irregularity. Therefore, ECD in eyes with irreversible edema due to endothelial decompensation was defined as 300 cells/mm^2^ as previously reported (7, 17, 19). To evaluate the association between postoperative ECD and risk factors, we analyzed ECD as absolute ECD and percentage of 24-month ECD loss (%ECD loss = [24-month ECD – graft ECD]/graft ECD × 100) (10). Graft survival periods were defined as from DSAEK surgery to the date of the clinical visit when irreversible corneal edema refractory to subsequent topical steroid use was noted. Graft survival time or time to censor (end of the study/loss of contact/withdrawal from the study) was calculated as the number of days between the date of DSAEK surgery and endothelial failure or censor. Three eyes of three patients with a follow-up period of < 1 month were excluded. We treated 13 eyes involving graft rejection episodes, four eyes with ocular infections, and nine eyes in which the procedure failed because of ocular surface complications or irregular graft thickness as censors. A total of 584 eyes of 482 patients were finally included. We selected the following 15 variables based on previous studies and our knowledge of donor-related factors (7–13) that can potentially affect graft survival and reduction in ECD after DSAEK: donor age, sex, domestic/imported graft, history of diabetes mellitus, cigarette smoking, alcohol consumption, drug abuse, history of laser-assisted *in situ* keratomileusis, lens status (phakic/intraocular lens [IOL]), cause of death (cardiac disease/cancer/cerebrovascular accident/respiratory disease/other diseases), refrigerated body/eyes, time from death to the preservation, time from death to operation, ECD, and graft endothelial folds (none/mild/moderate). The graft endothelial fold severity grade for each patient was determined based on slit-lamp microscopic examination findings before DSAEK. Briefly, grafts without folds were defined as having “no graft folds,” “mild graft folds” were defined by the presence of graft folds limited to <25% of the area of the total cornea, and “moderate graft folds” were defined by the presence of graft folds occupying more than 25% of the area of the total cornea. Among recipient/surgical factors, we evaluated age, sex, etiologies of bullous keratopathy, lens status at DSAEK, simultaneous cataract surgery, re-bubbling, central graft thickness, and graft diameter. Thus, a total of 23 potential preoperative risk factors (15 donor-related and eight recipient-related factors) were evaluated (Table 1).

**Table 1:** Demographics of all samples (N = 584).

**Donor and donor cornea characteristics**	
Donor age, median (IQR), range, y	66.0 (58, 72), 18–96
Donor sex, male, *n* (%)	373 (63.9)
Imported graft, *n* (%)	427 (73.1)
History of diabetes mellitus, *n* (%)	140 (24.0)
Cigarettes smoking, *n* (%)	323 (55.3)
Alcohol consumption, *n* (%)	206 (35.3)
Drug abuse, *n* (%)	73 (12.5)
History of LASIK, *n* (%)	34 (5.8)
Donor lens status, *n* (%)	
Phakic	487 (84.0)
IOL	97(16.6)
Cause of death, *n* (%)	
Cardiac disease	148 (25.3)
Cancer	147 (25.2)
Cerebrovascular accident	86 (14.8)
Respiratory disease	99 (17.0)
Others	104 (17.7)
Refrigerated/on ice, *n* (%)	343 (58.7)
Time from death to the preservation, median (IQR), range, h	8.0 (5.9, 11.9), 1.8–26.9
Time from death to operation, median (IQR), range, d	6.9 (6.0, 7.7), 2.0–9.6
Graft ECD, median (IQR), range, cells/mm^2^	2,637 (2,415, 2,921), 2,010–3,812
Endothelial folds, *n* (%)^*^	
None	157 (26.9)
Mild to moderate	427 (73.1)
**Recipient and surgical characteristics**	
Recipient age at DSAEK, median (IQR), range, y	73.5 (67, 79), 15–99
Recipient sex, male, *n* (%)	208 (35.6)
Etiology, *n* (%)	
FECD	59 (10.1)
LIBK	192 (32.9)
PBK	137 (23.5)
Regraft^†^	96 (16.4)
Others	100 (17.1)
Recipient lens status at DSAEK, *n* (%)	
Phakic	244 (41.8)
IOL/Aphakic	340 (58.2)
Simultaneous cataract surgery, *n* (%)	189 (32.4)
Re-bubbling, *n* (%)	79 (13.5)
Central graft thickness, median (IQR), range, μm*^‡^*	143 (123, 161), 53–270
Graft diameter, *n* (%), mm	
6.75–7.75	144 (24.7)
8.0–8.75	440 (75.3)

*DSAEK, Descemet's stripping automated endothelial keratoplasty; ECD, endothelial cell density; FECD, Fuchs' endothelial corneal dystrophy; IOL, intraocular lens; IQR, interquartile range; LASIK, laser-assisted in situ keratomileusis; LIBK, laser-iridotomy-related bullous keratopathy; PBK, pseudophakic bullous keratopathy*.

* *Grafts without folds were defined as having “no graft folds,” “mild graft folds” were defined by the presence of graft folds limited to <25% of the area of the total cornea, and “moderate graft folds” were defined by the presence of graft folds that occupied more than 25% of the area of the total cornea*.

† *Regraft is included in re-DSAEK (56 eyes), post penetrating keratoplasty (36 eyes), post-deep anterior lamellar keratoplasty (2 eyes) and post-Descemet's membrane endothelial keratoplasty (2 eyes)*.

‡ *Central graft thickness data was obtained in 503 eyes*.

### Statistical Analysis

The required sample size was based on previous studies (5, 7–13) which estimated that approximately 10% of grafts used DSAEK develop graft failure postoperatively. Given approximately 30% of withdrawal from the study, we determined that a sample size of at least 521 would provide 80% power to detect a difference in a hazard ratio of 0.60 or greater at an alpha value of 0.05 (two-sided). The cumulative probability of graft survival at the 2-year follow-up was calculated using the Kaplan–Meier method. In survival analysis, the collected categorical data were transformed into dummy and continuous variables and dichotomized with the median for use as categorical data. Recipient etiologies were investigated under non-FECD groups. A log-rank test was used to assess the association of each baseline factor with endothelial failure in univariate analysis. Factors with *P* < 0.05 in univariate analysis underwent proportional hazard analyses by log-log plot and Schoenfeld residuals tests and were included in multivariate Cox proportional hazard regression analysis for estimation of the independent predictors of endothelial failure in all patients. The prognostic model was prepared by combining the extracted risk factors (20, 21). Cumulative probability of graft survival was compared between the grafts with no risk factors and grafts with one or two risk factors. The Kaplan–Meier curves were plotted with non-FECD.

To evaluate the associations between postoperative ECD changes and risk factors adjusted for the effects of patient and surgeon factors, we used linear mixed effect models with random intercepts for recipient and surgeon effect. In the univariate model, the interaction of all potential risk factors and postoperative time were analyzed. Potential risk factors from univariate models with *P* < 0.10 were evaluated in a multivariate model, with %ECD loss after DSAEK. In the final model, the differences in postoperative ECD were compared between the grafts with no risk factors and grafts with one or two risk factors using a linear mixed effect model adjusted for etiology (fixed effect) and recipient and surgeon (random effect). Continuous variables were included in all ECD models in continuous form but were categorized for display in tables. Missing data were not imputed. Statistical analyses for graft survival were conducted using STATA/IC 16.0 for Windows (StataCorp LP, College Station, TX) and R version 4.3.0 for Windows (lme4 package, R Foundation for Statistical Computing, Vienna, Austria) was used for ECD analysis. All reported *P* values were 2-sided, and values < 0.05 were considered statistically significant.

## Results

### Demographics

In 584 eyes of 482 patients (Table 1), the median follow-up period was 24 months (interquartile range [IQR], 12–24 months). The recipients' age at DSAEK ranged from 15 to 99 years (median, 73.5 years), and 208 (35.6%) were male, while 582 were Asian (99.9%). The etiologies of BK included LIBK in 192 eyes (32.9%), PBK in 137 eyes (23.5%), regraft (re-DSAEK, 56 eyes [58.3%]; post-PKP, 36 eyes, [37.5%]; post-deep anterior lamellar keratoplasty, two eyes, [2.1%] and post-DMEK, two eyes, [2.1%]) in 96 eyes (16.4%), FECD in 59 eyes (10.1%), and other conditions (birth injury, chronic uveitis, endotheliitis, etc.) in 100 eyes (17.1%). The recipients' lens status at DSAEK was pseudophakic in 340 eyes (58.2%), and simultaneous DSAEK and cataract surgery was performed in 189 eyes (32.4%). Median central graft thickness was 143 μm (range, 53–270 μm) and the most common graft diameter was 8.0 mm in the current study. There were 373 (63.9%) male donors aged 18–96 years (median, 66.0 years). A total of 427 corneas (73.1%) were imported (157 corneas [26.9%] were domestic). A history of diabetes mellitus was present in 140 (24.0%) grafts, and the donor lens status was pseudophakic in 97 grafts (16.6%). The median donor ECD was 2,637 cells/mm^2^ (range, 2,010–3,812 cells/mm^2^), 157 grafts (26.9%) had no endothelial folds, while 427 grafts (73.1%) had mild to moderate endothelial folds before DSAEK.

### Graft Survival and Cox Proportional Hazards Analysis

**Figure 1:**
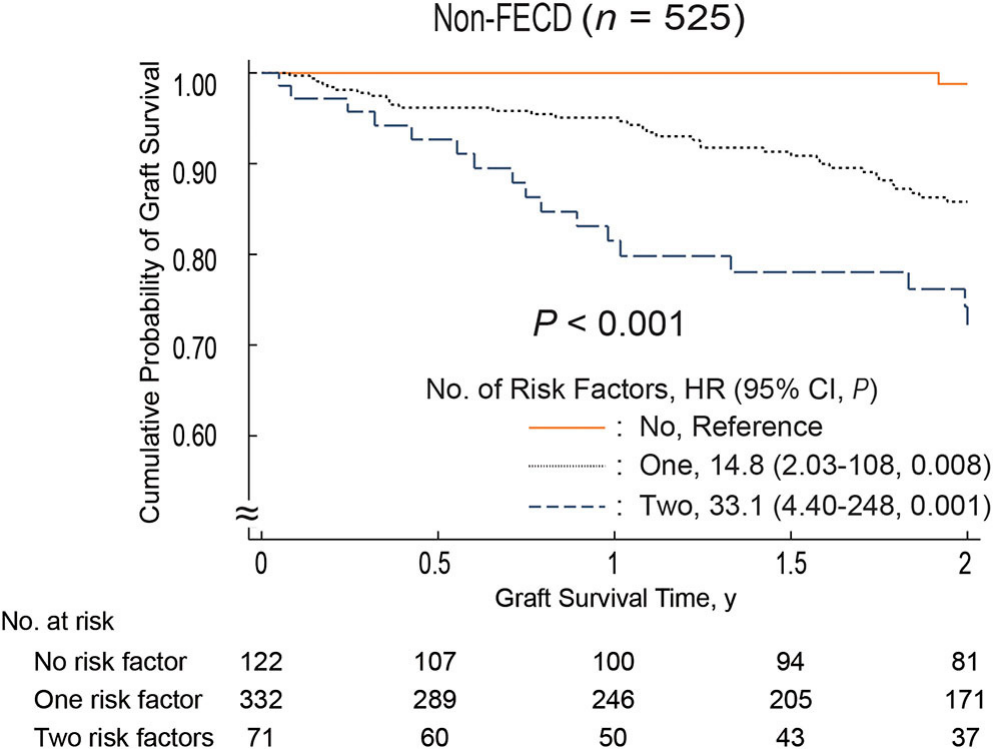
Two-year survival curves stratified patients based on graft-related risk factors. The risk factors are pseudophakic donor lens status and severe endothelial folds. Non-FECD eyes, graft survival was significantly better in eyes without graft-related risk factors compared to those with graft-related risk factors (*P* < 0.001; one risk factor: HR, 14.8; 95% CI, 2.03–108; *P* = 0.008; two risk factors: HR, 33.1; 95% CI, 4.40–248; *P* = 0.001). CI, confidence interval; FECD, Fuchs' endothelial corneal dystrophy; HR, hazard ratio.

During the 2-year follow-up period, endothelial failure occurred in 55 eyes (9.4%). The cumulative probability of endothelial failure after DSEAK in the entire cohort was 0.94 (95% confidence interval [CI], 0.91–0.96) at 1 year and 0.88 (95% CI, 0.84–0.91) at 2 years (Supplementary Figure 1). Among the 23 variables selected as potential preoperative risk factors and tested in a univariate analysis using the log-rank test, donor age (*P* = 0.04), history of diabetes mellitus (*P* = 0.007), donor lens status (*P* < 0.001), endothelial folds (*P* = 0.003), and etiology (*P* = 0.04) were found to be statistically significant. These five variables were added to a multivariate model. Cox proportional hazard regression analysis revealed that donor lens status (IOL, hazard ratio [HR], 2.67; 95% CI, 1.50–4.76; *P* = 0.001) and endothelial folds (mild to moderate, HR, 2.82; 95% CI, 1.20–6.62; *P* = 0.02) were risk factors associated with endothelial failure (Table 2). Figure 1 shows the influence of pseudophakic donor lens state and endothelial folds on graft survival in non-FECD eyes. In eyes with non-FECD, the outcome for grafts with one or two risk factors was significantly worse than that for those with no risk factor (one risk factor: HR, 14.8; 95% CI, 2.03–108; *P* = 0.008; two risk factors: HR, 33.1; 95% CI, 4.40–248; *P* = 0.001).

**Table 2:** Association between baseline factors and graft endothelial failure.

			**Log-rank test**	Multivariate models^*^	
**Prognostic factor**	***n***	**2-year graft survival (95% CI)**	***P***	**HR (95% CI)**	***P***
**Donor age, y**					
18–65	281	0.91 (0.86–0.94)	**0.04**	1 [reference]	0.48
66–96	303	0.85 (0.80–0.89)		1.23 (0.69–2.19)	
**Donor sex**					
Male	373	0.90 (0.86–0.93)	0.13		
Female	211	0.86 (0.79–0.90)			
**Imported graft**					
No	157	0.89 (0.85–0.92)	0.48		
Yes	427	0.87 (0.79–0.92)			
**History of diabetes mellitus**					
No	444	0.91 (0.87–0.93)	**0.007**	1 [reference]	0.09
Yes	140	0.81 (0.72–0.87)		1.63 (0.93–2.86)	
**Cigarettes smoking**					
No	323	0.87 (0.82–0.91)	0.42		
Yes	261	0.90 (0.84–0.93)			
**Alcohol consumption**					
No	378	0.87 (0.82–0.90)	0.22		
Yes	206	0.91 (0.85–0.94)			
**Drug abuse**					
No	511	0.89 (0.85–0.91)	0.55		
Yes	73	0.85 (0.72–0.92)			
**History of LASIK**					
No	550	0.88 (0.85–0.91)	0.92		
Yes	34	0.89 (0.70–0.96)			
**Donor lens status**					
Phakic	487	0.91 (0.88–0.94)	**<0.001**	1 [reference]	**0.001**
IOL	97	0.73 (0.62–0.81)		2.67 (1.50–4.76)	
**Cause of death**					
Cancer	147	0.87 (0.83–0.90)	0.22		
Non-cancer	437	0.93 (0.86–0.96)			
**Refrigerated/on ice**					
No	241	0.89 (0.84–0.93)	0.68		
Yes	343	0.88 (0.83–0.91)			
**Time from death to the preservation, h**					
1.8–7.9	287	0.89 (0.84–0.92)	0.49		
8.0–26.9	297	0.87 (0.82–0.91)			
**Time from death to operation, d**					
1.8–6.8	289	0.88 (0.82–0.91)	0.47		
6.9–9.6	295	0.89 (0.84–0.92)			
**Graft ECD, cells/mm** ^**2**^					
2,010–2,636	292	0.85 (0.80–0.89)	0.06		
2,637–3,812	292	0.91 (0.87–0.94)			
**Endothelial folds^†^**					
None	157	0.95 (0.89–0.98)	**0.003**	1 [reference]	**0.02**
Mild to moderate	427	0.86 (0.81–0.89)		2.82 (1.20–6.62)	
**Recipient age at DSAEK, y**					
15–72	259	0.87 (0.82–0.91)	0.56		
73–92	325	0.89 (0.84–0.92)			
**Recipient sex**					
Male	208	0.85 (0.78–0.90)	0.23		
Female	376	0.90 (0.86–0.93)			
**Etiology**					
FECD	59	0.98 (0.85–1.00)	**0.04**	1 [reference]	0.09
Non-FECD	525	0.87 (0.83–0.90)		5.50 (0.76–39.93)	
**Recipient lens status**					
Phakic	244	0.90 (0.84–0.93)	0.53		
IOL/Aphakic	340	0.88 (0.83–0.91)			
**Simultaneous CS**					
No	395	0.87 (0.83–0.90)	0.15		
Yes	189	0.92 (0.86–0.95)			
**Re-bubbling**					
No	505	0.88 (0.85–0.91)	0.18		
Yes	79	0.82 (0.67–0.91)			
**Central graft thickness, **μm**^‡^**					
53–130	167	0.85 (0.75–0.91)	0.93		
131–270	336	0.89 (0.84–0.92)			
**Graft diameter, mm**					
6.75–7.75	144	0.89 (0.85–0.91)	0.80		
8.00–8.75	440	0.89 (0.81–0.93)			

*CI, confidence interval; CS, cataract surgery; DSAEK, Descemet's stripping automated endothelial keratoplasty; ECD, endothelial cell density; FECD, Fuchs' endothelial corneal dystrophy; HR, hazard ratio; LASIK, laser-assisted in situ keratomileusis; IOL = intraocular lens*.

* *Cox proportional hazard regression analysis*.

† *Grafts without folds were defined as having “no graft folds,” “mild graft folds” were defined by the presence of graft folds limited to <25% of the area of the total cornea, and “moderate graft folds” were defined by the presence of graft folds occupying more than 25% of the area of the total cornea*.

*Bold numbers indicate P < 0.05*.

‡ *Central graft thickness data was obtained in 503 eyes*.

### Endothelial Cell Density Analysis

**Figure 2:**
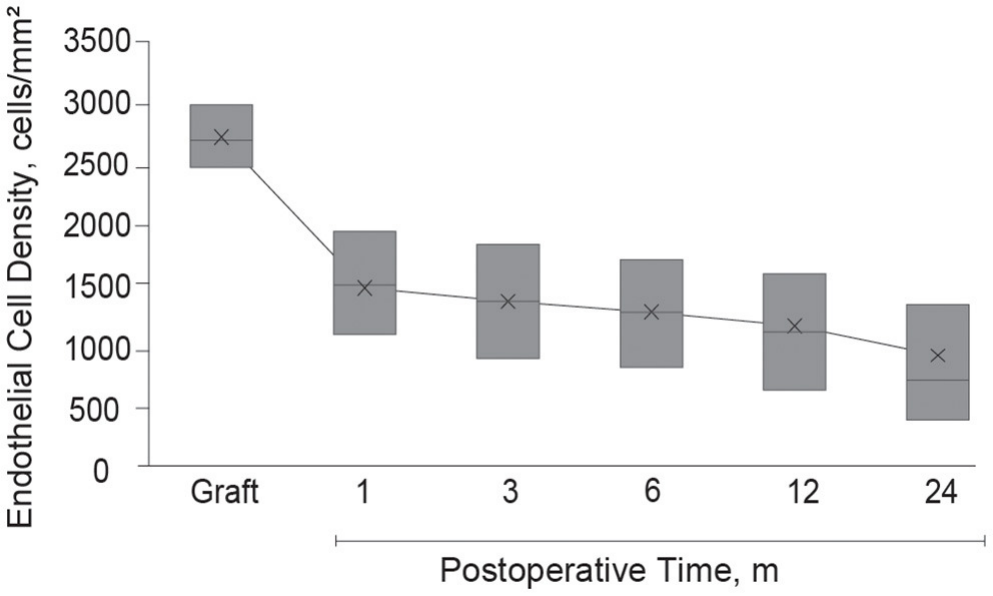
Endothelial cell density after Descemet's stripping automated endothelial keratoplasty. After Descemet's stripping automated endothelial keratoplasty, mean graft ECD decreased from 2,674 cells/mm^2^ (95% CI, 2,646–2,701) to 1,447 (95% CI, 1,377–1,516) at 1 month, 1,328 (95% CI, 1,269–1,387) at 3 months, 1,255 (95% CI, 1,198–1,311) at 6 months, 1,132 (95% CI, 1,076–1,190) at 12 months, and 904 (95% CI, 845–963) at 24 months (*P* < 0.001 at all time-points). The box represents interquartile range; the cross in each box is the mean; and the midline inside each box represents the median value. CI, confidence interval; ECD, endothelial cell density.

Endothelial images were obtained and analyzable in 332 eyes (56.8%) at 1 month, 415 eyes (71.1%) at 3 months, 442 eyes (75.6%) at 6 months, 454 eyes (77.7%) at 12 months, and 383 eyes (65.5%) at 24 months after DSAEK. Mean postoperative ECD was associated with postoperative time and decreased from 2,674 cells/mm^2^ (95% CI, 2,646–2,701) to 1,447 (95% CI, 1,377–1,516) at 1 month, 1,328 (95% CI, 1,269–1,387) at 3 months, 1,255 (95% CI, 1,198–1,311) at 6 months, 1,132 (95% CI, 1,076–1,190) at 12 months, and 904 (95% CI, 845–963) at 24 months (*P* < 0.001 at all timepoints, Figure 2). Factors associated with postoperative ECD reduction are shown in Table 3 and Supplementary Table 1. In a univariate model, donor age (*P* = 0.08), donor lens status (*P* = 0.02), graft ECD (*P* = 0.07), endothelial folds (*P* = 0.04), recipient sex (*P* = 0.05) and etiology (*P* = 0.06) had statistically significant association with postoperative ECD. Multivariable models showed that the risk factors independently associated with %ECD loss included donor lens status (*P* < 0.001), endothelial folds (*P* = 0.002) and etiology (*P* = 0.001). When the patients were stratified based on graft risk factors (pseudophakic donor lens state and endothelial folds), ECD after DSAEK was significantly greater in eyes receiving grafts from phakic eyes and without preoperative endothelial folds compared to those receiving grafts from pseudophakic eyes and/or with preoperative endothelial folds, in the mixed effect model adjusted with etiology (*P* = 0.007, Table 4).

**Table 3:** Factors associated with postoperative endothelial cell density.

		Univariate models^*^, **^†^**		Multivariate models^*^, **^‡^**	
**Prognostic factor**	***n***	**Mean ECD at 24-month (95% CI)**	***p***	**Mean %ECD loss at 24-month (95% CI)**	***p***
Donor age, y					
18–59	107	992 (883–1,100)	0.08	63.4 (59.6–67.3)	0.21
60–69	128	981 (870–1,092)		64.1 (60.3–68.0)	
70–79	116	767 (672–862)		70.7 (67.1–74.1)	
80–95	32	799 (577–1,022)		71.3 (64.0–78.7)	
Donor lens status					
Phakic	312	967 (900–1,033)	0.02	64.3 (61.9–66.6)	**<0.001**
IOL	71	628 (522–734)		76.5 (72.7–80.2)	
Graft ECD, cells/mm^2^					
2,010–2,499	111	669 (590–747)	0.07	70.8 (67.3–74.2)	0.08
2,500–2,749	123	845 (752–938)		67.4 (63.8–71.0)	
2,750–2,999	75	1013 (881–1,145)		64.7 (60.1–69.3)	
3,000–3,812	74	1244 (1,073–1,414)		60.5 (54.9–66.0)	
Endothelial folds^§^					
None	105	1069 (959–1,179)	0.04	60.2 (56.2–64.3)	**0.002**
Mild to moderate	278	842 (773–911)		68.9 (66.5–71.3)	
Recipient sex					
Male	133	828 (729–927)	0.05	69.4 (65.9–72.8)	0.07
Female	250	944 (871–1,018)		65.0 (62.4–67.6)	
Etiology					
FECD	35	1245 (1,043–1,448)	0.06	54.7 (47.8–61.6)	**0.001**
Non-FECD	348	867 (808–931)		67.7 (65.6–69.8)	

*CI, confidence interval; ECD, endothelial cell density; FECD, Fuchs' endothelial corneal dystrophy; IOL, intraocular lens*.

* *Adjusted for recipient and surgeon (random effect)*.

† *The interaction of potential risk factors and postoperative time were analyzed*.

‡ *The percentage ECD loss at 24-month is calculated as (24-month ECD – graft ECD)/graft ECD × 100*.

§ *Grafts without folds were defined as having “no graft folds,” “mild graft folds” were defined by the presence of graft folds limited to <25% of the area of the total cornea, and “moderate graft folds” were defined by the presence of graft folds occupying more than 25% of the area of the total cornea*. *Bold numbers indicate P < 0.05*.

**Table 4:** Endothelial cell density after Descemet's stripping automated endothelial keratoplasty by graft-related risk factors.

	No risk factor^*^		**One risk factor**		**Two risk factors**	
**Postoperative time**	***n***	**Mean (95% CI)**	***n***	**Mean (95% CI)**	***n***	**Mean (95% CI)**
Graft	108	2,717 (2,651–2,783)	321	2,677 (2,639–2,714)	155	2,638 (2,586–2,690)
1 month	70	1,639 (1,508–1,770)	180	1,461 (1,366–1,557)	82	1,251 (1107–1,395)
3 months	78	1,552 (1,429–1,674)	225	1,316 (1,237–1,395)	112	1,197 (1078–1,315)
6 months	83	1,507 (1,386–1,628)	238	1,258 (1,183–1,334)	121	1,074 (966–1,183)
12 months	88	1,454 (1,319–1,589)	244	1,114 (1,039–1,188)	122	940 (836–1,044)
24 months	73	1,166 (1,033–1,298)	200	931 (847–1,014)	110	682 (589–776)
*p* ^†^				0.007	

*CI, confidence interval*.

* *The risk factors are pseudophakic donor lens status and severe endothelial folds*.

† *Analyzed with a linear mixed-effect model adjusted for etiology (fixed effect) and recipient and surgeon (random effect)*.

## Discussion

We identified donor lens status and preoperative graft endothelial folds as risk factors associated with graft endothelial failure and ECD reduction after DSAEK. Furthermore, we demonstrated that these risk factors were clinically relevant especially in non-FECD and not in FECD, since these were associated with endothelial failure after DSAEK in non-FECD eyes. Although the prognosis of endothelial keratoplasty has been reported to be poor in eyes with PBK (8, 10, 12) especially in BK eyes after glaucoma surgery, in comparison with FECD (7, 12, 22), grafting is the only solution for such eyes with graft failure. Our results suggested that selecting better grafts with no endothelial folds or from phakic eyes for non-FECD patients may prolong graft survival after DSAEK. Our results also indicated that differences in imported or domestic donors did not have an adverse influence on endothelial failure after DSAEK.

Corneal transplants are performed in 116 countries, and imported grafts are used in 70 countries (23). The international organ-sharing program for corneal transplants has successfully grown because corneal tissue can be preserved for more than 1 week (24). Thus, understanding the donor-related risk factors from the global/transnational perspective and optimization of corneal donor tissues is important for both exporting and importing countries.

Previous studies identified several factors, including lower graft ECD (10, 12), history of glaucoma surgery (12, 13, 25), preoperative diagnosis (8, 10, 12), presence of donor diabetes mellitus (8, 10), preservation time of donor tissues (11), pre-existing iris damage (7), age (13, 26), race (4), sex (22, 26), graft size (7, 27), and pre-lamellar dissection corneal thickness (9), as risk factors for graft failure and/or rapid ECD loss after DSAEK. In contrast, long-term graft survival is known to be greater in eyes with relatively healthy peripheral endothelial cells (i.e., FECD/keratoconus) than those with BK (28, 29). Endothelial cells may migrate from the donor to the recipient eyes without peripheral endothelium (i.e., non-FECD), resulting in lower graft ECD and earlier graft failure, whereas ECD is greater in the periphery than in the center and peripheral regions of the cornea and cell migration from the host to the donor graft have been documented in corneal transplants (27, 29, 30). In the current study, we revealed that graft survival in eyes with non-FECD was significantly greater in eyes from donors with a phakic eye (HR: 2.67) and no endothelial folds (HR: 2.82). Furthermore, postoperative ECD decrease was significantly associated with donor lens status (IOL) and endothelial folds.

We identified pseudophakic donor lens status as a risk factor associated with ECD reduction and graft de-compensation after DSAEK, in contrast, the recipients' lens status did not show a statistically significant difference. This may be related to the relatively short follow-up period of 2 years after DSAEK. The annual ECD reduction rate in normal eyes is 0.9%, which can increase up to 2.5% per year after cataract surgery (31). Kawai et al. reported elevated levels of inflammatory cytokines such as interleukin-8 and monocyte-chemotactic protein-1 after cataract surgery (32). Our recent prospective studies have shown that an aqueous humor (AqH) microenvironment with elevated levels of inflammatory cytokines is associated with rapid loss of ECD after PKP and DSAEK (17, 18, 25, 33, 34). Our multi-omics analyses of human corneal endothelial cells identified stress-induced cell senescence as an upregulated biological process in BK (35). Collectively, these results suggest that the pseudophakic donor corneal endothelium shows deterioration in quality, such as cell aging or vulnerability to the pathological microenvironment in the AqH, that potentially leads to rapid ECD loss and endothelial failure after DSAEK.

In the current study, the preoperative presence of graft folds was associated with endothelial failure and lower ECD after DSAEK. Previous studies have reported the existence of dead cells in donor corneal endothelium preserved in Optisol-GS and stored at a temperature between 4° and 8°C before transplantation (36). Corneal folding has been shown to be significantly correlated with a reduction in corneal endothelial cells, and various studies using cell staining techniques have observed a higher concentration of dead/apoptotic endothelial cells along areas with corneal folds (37, 38). We checked the ECD before surgeries in all donor grafts, but the area of ECD measurement is very limited, approximately 0.24 × 0.35 mm (39), and ECD cannot be measured in the area with endothelial folds. A series of these studies suggested the difference between ECD values obtained by eye bank specular microscopy and the actual viable endothelial cells on the donor graft. The origin/mechanism of donor corneal folds has not been closely explored (40). In our sub-analysis, we found that donor age and time from death to the preservation were associated with the severity grade of the graft endothelial folds (Supplementary Table 2). There was no statistically significant difference between fold severe levels (mild folds *vs*. moderate folds) in endothelial failure after DSAEK (*p* = 0.91, data not shown). Further studies are necessary to evaluate the association between the presence of graft folds and reduction in viable corneal endothelial cells. Other potential risk factors discussed in previous studies (2, 4, 7–13, 22, 26, 27) were not associated with endothelial failure in this study: such as donor age (*P* = 0.48, in graft survival analysis), donor sex (*P* = 0.13), history of diabetes mellitus (*P* = 0.09), graft ECD (*P* = 0.06), gender matching (*P* = 0.12, data not shown), re-bubbling (*P* = 0.18), graft thickness (*P* = 0.93), and graft diameter (*P* = 0.80). This may have reflected differences in the cohort of recipients.

This study had some limitations. First, heterogeneous etiologies, such as FECD, PBK, LIBK, and regraft, could potentially have caused bias. We found that pseudophakic donor lens status/preoperative endothelial folds were risk factors for poor prognosis of DSAEK in the non-FECD group. Further analysis that stratified the patients based on etiology for BK was attempted. DSAEK using a graft from pseudophakic donor eyes with preoperative endothelial folds showed a trend of poor prognosis in a non-FECD group, but there was no statistically significant difference for BK other than LIBK (Supplementary Figure 2). A larger sample size and longer follow-up period are needed to further assess risk factors and donor-recipient matching. Second, almost all the subjects in this study were Japanese, and future studies will be needed to substantiate the results in other populations. Third, we could not identify the exact pathological mechanism involved in ECD loss and endothelial failure in eyes with these risk factors. We recently showed that pathological alterations in the microenvironment of AqH due to iris damage predisposed to ECD loss via exacerbated stress-induced cell senescence (35).

In conclusion, grafts from pseudophakic donor eyes with preoperative endothelial folds are identified as risk factors for endothelial failure after DSAEK in recipients with non-FECD. The results of this study suggest that optimization of corneal donor tissues for patients undergoing endothelial keratoplasty is important, especially for non-FECD patients.

## Data Availability Statement

The raw data supporting the conclusions of this article will be made available by the authors, without undue reservation.

## Ethics Statement

The studies involving human participants were reviewed and approved by the Institutional Ethics Reviewer Board of Tokyo Dental College Ichikawa General Hospital (Acceptance No. I 18-19). The patients/participants provided their written informed consent to participate in this study.

## Author Contributions

SN and TY had full access to all the data in the study and take responsibility for the integrity of the data and the accuracy of the data analysis, study concept and design, and drafting of the manuscript. SN, TY, MH, HN, JS, and KH critical revision of the manuscript for important intellectual content. SN, TY, and HN statistical analysis. JS and SN obtained funding. JS, TY, and MH study supervision. All authors acquisition, analysis, or interpretation of data and administrative, technical, or material support.

## Funding

This study received funding from a Novartis Research Grant. The funder was not involved in the study design, collection, analysis, interpretation of data, the writing of this article or the decision to submit it for publication.

## Conflict of Interest

The authors declare that the research was conducted in the absence of any commercial or financial relationships that could be construed as a potential conflict of interest.

## Publisher's Note

All claims expressed in this article are solely those of the authors and do not necessarily represent those of their affiliated organizations, or those of the publisher, the editors and the reviewers. Any product that may be evaluated in this article, or claim that may be made by its manufacturer, is not guaranteed or endorsed by the publisher.

## Meeting Presentation

Presented at the Japan Cornea Conference, Tokyo, Japan, April 2020.
