# Supplementary material for: Donor-Related Risk Factors for Graft Decompensation Following Descemet's Stripping Automated Endothelial Keratoplasty
Source: Front Med (Lausanne). 2022 Feb 4;9:810536. doi: 10.3389/fmed.2022.810536 (PMC8889573; doi:10.3389/fmed.2022.810536)
Supplement: Supplementary file 1 [file Data_Sheet_1.PDF]

Supplementary Table 1. Factors Not Associated with Postoperative Endothelial Cell Density

	Univariate Models*				
**Prognostic Factor**	***n***	**Graft ECD (95% CI)**	***n***	**Mean ECD at 24-month (95% CI)**	***p***
Donor sex					
Male	373	2683 (2649-2717)	244	941 (866-1017)	.81
Female	211	2657 (2610-2705)	139	838 (744-933)	
Imported graft					
No	157	2848 (2798-2898)	103	972 (845-1094)	.74
Yes	427	2610 (2579-2640)	280	879 (812-947)	
History of diabetes mellitus					
No	444	2682 (2651-2714)	286	950 (880-1021)	.49
Yes	140	2646 (2590-2703)	97	767 (662-872)	
Cigarettes smoking					
No	323	2716 (2679-2753)	214	878 (801-954)	.27
Yes	261	2621 (2581-2661)	169	937 (844-1030)	
Alcohol consumption					
No	378	2727 (2687-2757)	255	916 (840-991)	.88
Yes	206	2586 (2544-2627)	128	881 (786-976)	
Drug abuse					
No	511	2677 (2648-2707)	339	903 (841-965)	.87
Yes	73	2649 (2573-2726)	44	914 (719-1109)	
History of LASIK					
No	550	2677 (2649-2706)	361	902 (841-962)	.74
Yes	34	2614 (2525-2704)	22	946 (673-1218)	
Cause of death					
Cancer	147	2720 (2666-2774)	93	963 (831-1094)	.69
Non-cancer	437	2658 (2626-2690)	290	885 (819-951)	
Refrigerated/on ice					
No	241	2777 (2735-2818)	163	932 (838-1026)	.84
Yes	343	2601 (2567-2636)	220	883 (807-959)	
Time from death to the preservation, h					
1.8-4.9	98	2715 (2649-2781)	66	932 (781-1083)	.86
5.0-7.9	189	2626 (2576-2676)	122	910 (806-1014)	
8.0-11.9	155	2685 (2631-2739)	98	834 (717-951)	
12.0-26.9	142	2696 (2642-2749)	97	949 (830-1067)	
Time from death to operation, d					
1.8-4.9	72	2821 (2747-2895)	51	983 (806-1160)	.19
5.0-6.9	228	2644 (2599-2689)	146	910 (810-1009)	
7.0-7.9	165	2648 (2600-2695)	105	922(808-1036)	
8.0-9.6	119	2678 (2614-2742)	81	821 (706-936)	

Supplementary Table 1. Factors Not Associated with Postoperative Endothelial Cell Density (continued)

	Univariate Models*				
**Prognostic Factor**	***n***	**Graft ECD (95% CI)**	***n***	**Mean ECD at 24-month (95% CI)**	***p***
Recipient age at DSAEK, y					
18-65	118	2715 (2655-2776)	85	916 (802-1030)	
66-75	235	2685 (2643-2728)	172	895 (802-987)	.98
76-92	231	2641 (2595-2686)	126	909 (804-1013)	
Recipient lens status at DSAEK					
Phakic	244	2655 (2614-2697)	158	989 (893-1086)	.11
IOL/Aphakic	340	2687 (2650-2724)	225	844 (770-918)	
Simultaneous CS					
No	395	2684 (2650-2718)	264	867 (795-939)	.10
Yes	189	2652 (2606-2699)	119	986 (883-1090)	
Re-bubbling					
No	505	2676 (2647-2706)	330	945 (881-1009)	.25
Yes	79	2657 (2579-2734)	53	648 (517-780)	
Central graft thickness, µm					
53-130	167	2622 (2571-2673)	101	899 (783-1015)	.37
131-270	336	2665 (2630-2701)	225	918 (838-998)	
Graft diameter, mm					
6.75 to 7.75	144	2719 (2666-2774)	96	820 (712-928)	.15
8.00 to 8.75	440	2659 (2627-2691)	287	932 (862-1002)	

CI = confidence interval; CS = cataract surgery; DSAEK = Descemet's stripping automated endothelial keratoplasty; ECD = endothelial cell density; IOL = intraocular lens; LASIK = laser-assisted in situ keratomileusis.

** The interaction of potential risk factors and postoperative time were analyzed with a linear mixed-effect model adjusting for recipient and surgeon (random effect).*

Supplementary Table 2. Association Between Baseline Factors and Preoperative Endothelial Folds

Prognostic Factor	*n*	Folds, Mild to Moderate, *n* (%)*	Chi-squared Test *P*	Multivariate Models† OR (95% CI)	Multivariate Models† *P*
Donor age, y					
18-65	281	190 (67.6)	**.004**	1 [reference]	**.02**
66-96	303	237 (78.2)		1.50 (1.08-2.09)	
Donor sex					
Male	373	272 (72.9)	.89		
Female	211	155 (73.5)			
Imported graft					
No	157	109 (68.8)	.15		
Yes	427	319 (74.7)			
History of diabetes mellitus					
No	444	324 (73.0)	.89		
Yes	140	103 (73.6)			
Cigarettes smoking					
No	323	239 (74.0)	.60		
Yes	261	188 (72.0)			
Alcohol consumption					
No	378	277 (73.3)	.90		
Yes	206	150 (72.8)			
Drug abuse					
No	511	375 (73.4)	.70		
Yes	73	52 (71.2)			
History of LASIK					
No	550	406 (73.8)	.12		
Yes	34	21 (61.8)			
Donor lens status					
Phakic	487	350 (71.9)	.13		
IOL	97	77 (79.4)			
Cause of death					
Cardiac disease	148	116 (78.3)	.19		
Cancer	147	101 (68.7)			
CVA	86	68 (79.1)			
Respiratory disease	99	69 (69.7)			
Others	104	73 (70.2)			
Refrigerated/on ice					
No	241	171 (71.0)	.32		
Yes	343	256 (74.6)			
Time from death to the preservation, h					
1.8-7.9	287	192 (66.9)	**.001**	1 [reference]	**< .001**
8.0-26.9	297	235 (79.1)		2.00 (1.37-2.92)	

Supplementary Table 2. Association Between Baseline Factors and Preoperative Endothelial Folds (continued)

Prognostic Factor	*n*	Folds, Mild to Moderate, *n* (%)*	Chi-squared Test *P*	Multivariate Models† OR (95% CI)
Time from death to operation, d				
1.8-6.8	289	208 (72.0)	.54	
6.9-9.6	295	219 (74.2)		
Graft ECD, cells/mm^2				
2010-2636	292	213 (73.0)	.93	
2637-3812	292	214 (73.3)		

CI = confidence interval; CVA = Cerebrovascular accident; DSAEK = Descemet's stripping automated endothelial keratoplasty; ECD = endothelial cell density; OR = odds ratio; LASIK = laser-assisted in situ keratomileusis; IOL = intraocular lens.

** Grafts without folds were defined as having "no graft folds", "mild graft folds" were defined by the presence of graft folds limited to less than 25% of the area of the total cornea, and "moderate graft folds" were defined by the presence of graft folds occupying more than 25% of the area of the total cornea.*

† Logistic regression analysis.

Bold numbers indicate P < .05.

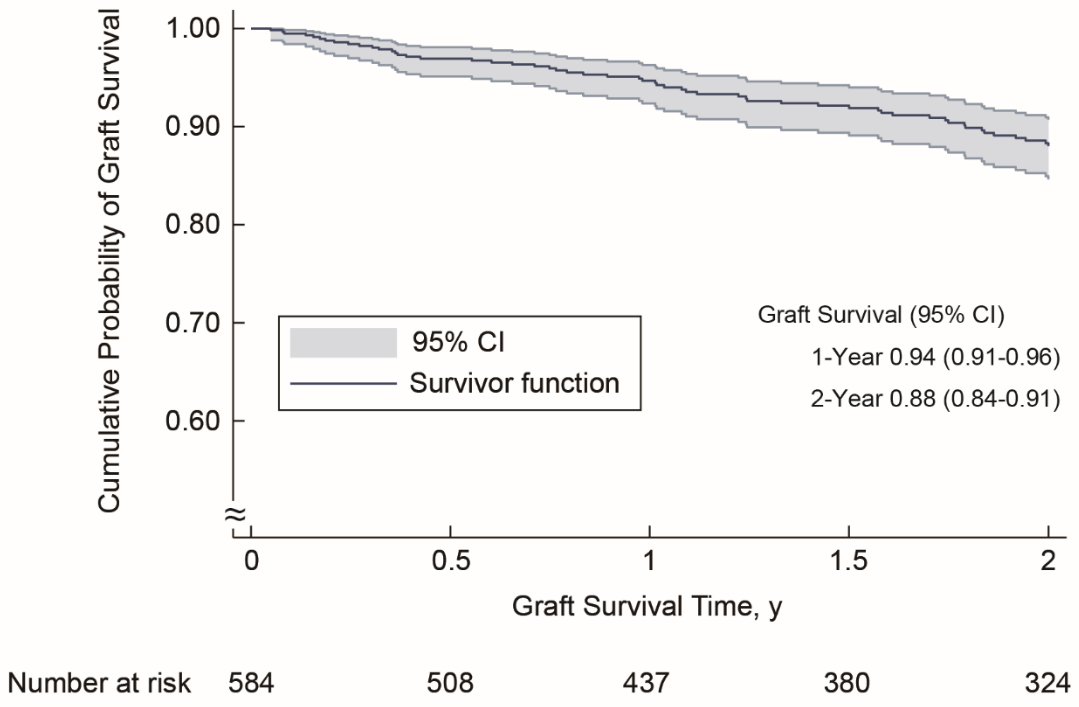

Supplementary Figure 1. Kaplan–Meier survival curve of all grafts. The cumulative probability of endothelial failure after Descemet's stripping automated endothelial keratoplasty in the entire cohort was 0.94 (95% CI, 0.91-0.96) at 1 year and 0.88 (0.84-0.91) at 2 years. The gray area represents the 95% CI for the probability estimate with vertical inflections indicating censored data. CI = confidence interval.

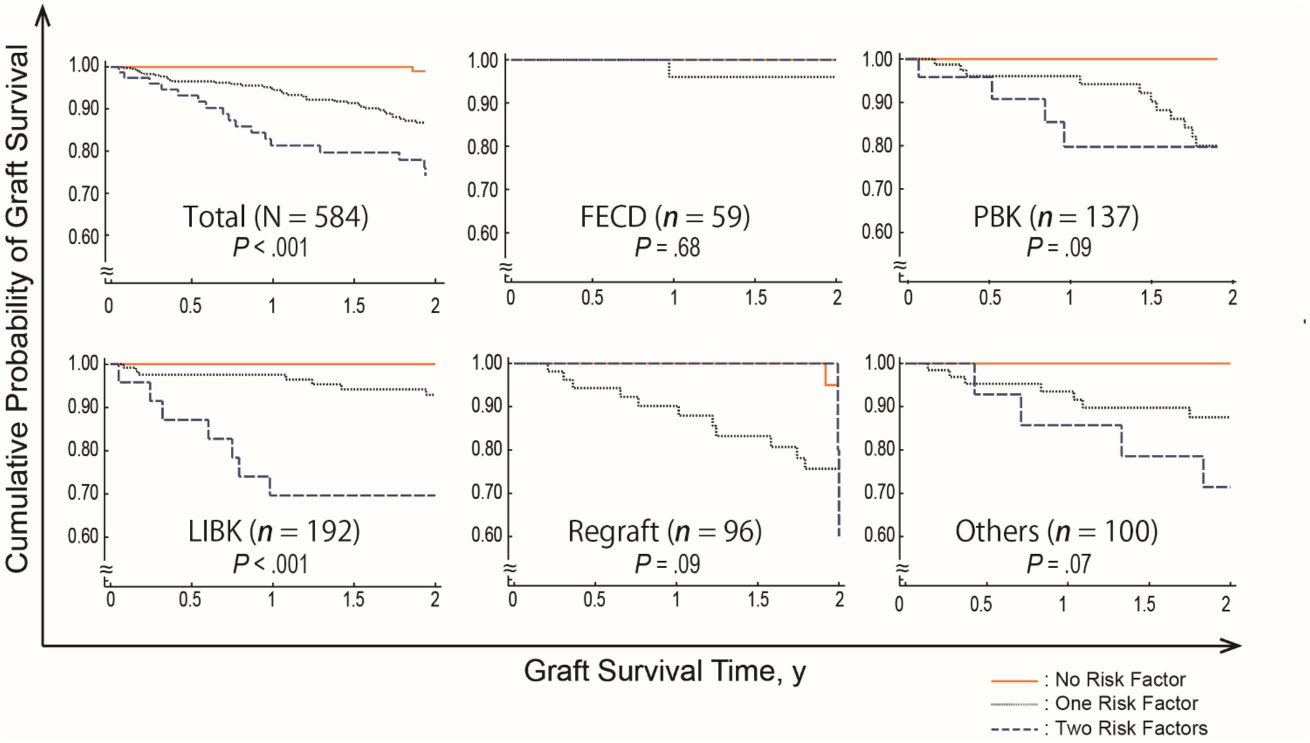

Supplementary Figure 2. Two-year survival curves stratified patients based on etiology and graft-related risk factors. The risk factors are pseudophakic donor lens status and severe endothelial folds. FECD = Fuchs' endothelial corneal dystrophy; LIBK = laser-iridotomy-related bullous keratopathy; PBK = pseudophakic bullous keratopathy.
